# International findings with the Achenbach System of Empirically Based Assessment (ASEBA): applications to clinical services, research, and training

**DOI:** 10.1186/s13034-019-0291-2

**Published:** 2019-07-05

**Authors:** Thomas M. Achenbach

**Affiliations:** Department of Psychiatry, University of Vermont, 1 South Prospect Street, Burlington, VT 05401 USA

**Keywords:** Multicultural, ASEBA, Norms, Multi-informant, International, Mental health services

## Abstract

The purpose of this invited article is to present multicultural norms and related international findings obtained with the Achenbach System of Empirically Based Assessment (ASEBA) by indigenous researchers in over 50 societies. The article describes ASEBA instruments for which multicultural norms are available, plus procedures for constructing the multicultural norms. It presents applications to clinical services, including use of multi-informant data for assessing children and their parents. The Multicultural Family Assessment Module (MFAM) enables mental health providers to view side-by-side bar graphs of child and parent scores on syndromes, DSM-oriented scales, Internalizing, Externalizing, and Total Problems. Evidence-based assessment of progress and outcomes is facilitated by the Progress & Outcomes App (P&O App). Research applications are outlined, including longitudinal and outcomes research. Applications to training mental health providers include having trainees study standardized multi-informant assessment data prior to interviewing children and their parents. Trainees can also sharpen their clinical skills by completing assessment forms to describe children and their parents, and then using ASEBA software to compare their ratings with ratings by children, parents, and other informants. Practical evidence-based assessment instruments with multicultural norms enable mental health providers, researchers, and trainees to perform intake, progress, and outcome assessments of children and their parents in terms of a standardized international clinical data language.

## Background

This article was invited by CAPMH Editor Joerg Fegert. Its purpose is to present multicultural norms and related international findings obtained with the Achenbach System of Empirically Based Assessment (ASEBA) by indigenous researchers in over 50 societies from every inhabited continent. The article describes ASEBA instruments for which multicultural norms are available and procedures for constructing the multicultural norms. It presents applications to clinical services, including use of multi-informant data for assessing children and their parents. The Multicultural Family Assessment Module (MFAM) enables mental health providers to view side-by-side bar graphs of parent and child scores on syndromes, DSM-oriented scales, Internalizing, Externalizing, and Total Problems. Evidence-based assessment of progress and outcomes is facilitated by the Progress & Outcomes App (P&O App). Research applications are outlined, including longitudinal and outcomes research. Applications to training mental health providers include having trainees study standardized multi-informant assessment data prior to interviewing parents and children. Trainees can also sharpen their clinical skills by completing assessment forms to describe parents and children and then using ASEBA software to compare their completed forms with forms completed by parents and youths.

## Main text

The ASEBA includes standardized assessment instruments for obtaining self- and collateral-reports of behavioral, emotional, social, and thought problems and strengths manifested by people from age 1½ to 90+ years. The ASEBA also includes instruments for assessing children’s functioning during clinical interviews and during individual ability and achievement tests [1], which are not addressed in this article. The self- and collateral-report instruments are tailored to assessment of people at ages 1½–5, 6–18, 18–59, and 60–90+ and to the kinds of informants who are appropriate for the assessed person’s age.

The purpose of this article is to present multicultural norms and related international findings obtained by collaborating indigenous researchers in over 50 societies from every inhabited continent. (“Societies” refer to geopolitically demarcated populations having a dominant language, including countries but also distinctive populations that do not comprise countries, such as Hong Kong, Puerto Rico, and Flanders—the Flemish-speaking region of Belgium.) The main focus will be on ages 1½–18, for which “children” will be used. However, because parents and other adults must be involved in efforts to help children, multicultural aspects of adult assessment will also be addressed. After international findings are presented, applications to clinical services, research, and training will be outlined.

### ASEBA instruments having multicultural norms

The ASEBA instruments for which multicultural norms have been constructed are standardized forms that include items that describe a broad spectrum of problems. Informants rate the problem items as *0 *=* not true (as far as you know), 1 *=* somewhat or sometimes true,* or *2 *=* very true or often true* over periods specified on the forms, such as 2 months or 6 months.

The problem items are worded to be easily understood by the kinds of informants for whom they are intended. As an example, the Child Behavior Checklist for Ages 6–18 (CBCL/6–18) is designed to be completed by parent figures who are asked to provide 0–1–2 ratings of items such as *Acts too young for age; Can’t concentrate, can’t pay attention for long; Cruel to animals; Gets in many fights; Unhappy, sad, or depressed;* and *Worries.* The items have been selected and refined through many iterations of testing with clinical and population samples to assess problems that are found to be significantly associated with clinical status and that are well-understood by the intended informants. Most of the forms also include items for assessing various kinds of strengths. The forms can be self-administered online or on paper or can be administered by interviewers without specialized training. Table 1 lists the ASEBA forms addressed in this article, while Table 2 lists languages in which translations of the forms are available.

**Table 1:** Self- and collateral-assessment instruments having multicultural norms

Age ranges	Instruments	Informants
1½–5	Child Behavior Checklist for Ages 1½–5 (CBCL/1½–5)	Parent figures
	Caregiver–Teacher Report Form (C-TRF)	Daycare providers; preschool teachers
6–18	Child Behavior Checklist for Ages 6–18 (CBCL/6–18)	Parent figures
	Teacher’s Report Form (TRF)	Teachers; school counselors
11–18	Youth Self-Report (YSR)	Youths
18–59	Adult Self-Report (ASR)	Adults
	Adult Behavior Checklist (ABCL)	Adult collaterals
60–90+	Older Adult Self-Report (OASR)	Older adults
	Older Adult Behavior Checklist (OABCL)	Older adult collaterals

**Table 2:** Translations of ASEBA forms

1. Afaan Oromo (Ethiopia)	36. Georgian	71. Polish
2. Afrikaans	37. German	72. Portuguese (Angola, Portugal)
3. Albanian/Kosova	38. Greek	73. Portuguese (Brazilian)
4. American Sign Language	39. Gujarati (India)	74. Portuguese Creole
5. Amharic (Ethiopia)	40. Haitian Creole	75. Punjabi (India)
6. Arabic	41. Hebrew	76. Romanian
7. Armenian	42. Hindi (India)	77. Russian
8. Auslan (Australian Sign Language)	43. Hungarian	78. Sami (Norway)
9. Bahasa (Indonesia)	44. Icelandic	79. Samoan
10. Bahasa (Malaysia)	45. Italian	80. Sepedi (Northern Sotho)
11. Bangla (Bangladesh)	46. Japanese	81. Serbian
12. Basque (Spain)	47. Kannada (India)	82. Sesotho (Southern Sotho)
13. Bemba (Zambia)	48. Khmer (Cambodia)	83. Sinhala (Sri Lanka)
14. Bengali (India)	49. Kiembu (Kenya)	84. Slovak
15. Bosnian	50. Kikamba (Kenya)	85. Slovene
16. Braille	51. Kiswahili (Kenya)	86. Somali
17. British Sign Language	52. Korean	87. Spanish (Castilian)
18. Bulgarian	53. Laotian	88. Spanish (Latino)
19. Burmese (Myanmar)	54. Latvian	89. Swahili
20. Catalan (Spain)	55. Lithuanian	90. Swedish
21. Cebuano (Philippines)	56. Luganda (Uganda)	91. Tagalog (Philippines)
22. Chinese	57. Luo (Uganda)	92. Tamil (India)
23. Croatian	58. Macedonian	93. Telugu (India)
24. Czech	59. Malayalam (India)	94. Thai
25. Danish	60. Maltese	95. Tigrinya (Eritrea)
26. Dutch (Netherlands, Flanders)	61. Manipuri (India)	96. Tibetan
27. Estonian	62. Marathi (India)	97. TshiVenda (South Africa)
28. Farsi/Persian (Iran)	63. Mauritian Creole	98. Turkish
29. Finnish	64. Montenegrin	99. Ukrainian
30. Flemish	65. Nepalese	100. Urdu (India, Pakistan)
31. French (Belgian)	66. Norwegian	101. Vietnamese
32. French (Canadian)	67. Nyanja (Zambia)	102. Visayan (Philippines)
33. French (Parisian)	68. Omoro (Ethiopia)	103. Xhosa (South Africa)
34. Ga (Ghana)	69. Papiamento (Curacao)	104. Zulu
35. Galician (Spain)	70. Pashto (Afghanistan, Pakistan)	

Languages into which at least one ASEBA form has been translated. Please visit http://www.aseba.org for updated lists of translations of each ASEBA form

### Testing empirically derived syndromes in multiple societies

The problem items of the forms listed in Table 1 have been factor analyzed to identify syndromes of problems that tend to co-vary in ratings by each kind of informant for a particular age range. This constitutes a “bottom-up” approach to constructing taxonomies of psychopathology based on ratings of large samples of individuals on each form. The initial factor analyses were done on ratings for Anglophone populations, mainly in the US. However, to test the generalizability of the syndromes to other societies, the syndromes derived from Anglophone samples were used as models in confirmatory factor analyses (CFAs) of ratings of population samples from dozens of other societies [11–19, 24].

The CFA findings have supported the syndromes derived from Anglophone samples in all societies analyzed to date. Although it is possible that problem items not included on the ASEBA forms and/or other analytic methods might reveal additional syndromes in some societies, the following six syndromes derived from parent and caregiver/teacher ratings for ages 1½–5 have been supported in dozens of societies: *Emotionally Reactive, Anxious/Depressed, Somatic Complaints, Withdrawn, Attention Problems,* and *Aggressive Behavior.* An additional syndrome—designated as *Sleep Problems*—has also been supported for parent ratings. For ages 6–18, the following eight syndromes derived from parent-, teacher-, and youth self-ratings have been supported in dozens of societies: *Anxious/Depressed, Withdrawn/Depressed, Somatic Complaints, Social Problems, Thought Problems, Attention Problems, Rule*-*Breaking Behavior,* and *Aggressive Behavior.*

### Constructing multicultural norms

**Fig. 1:**
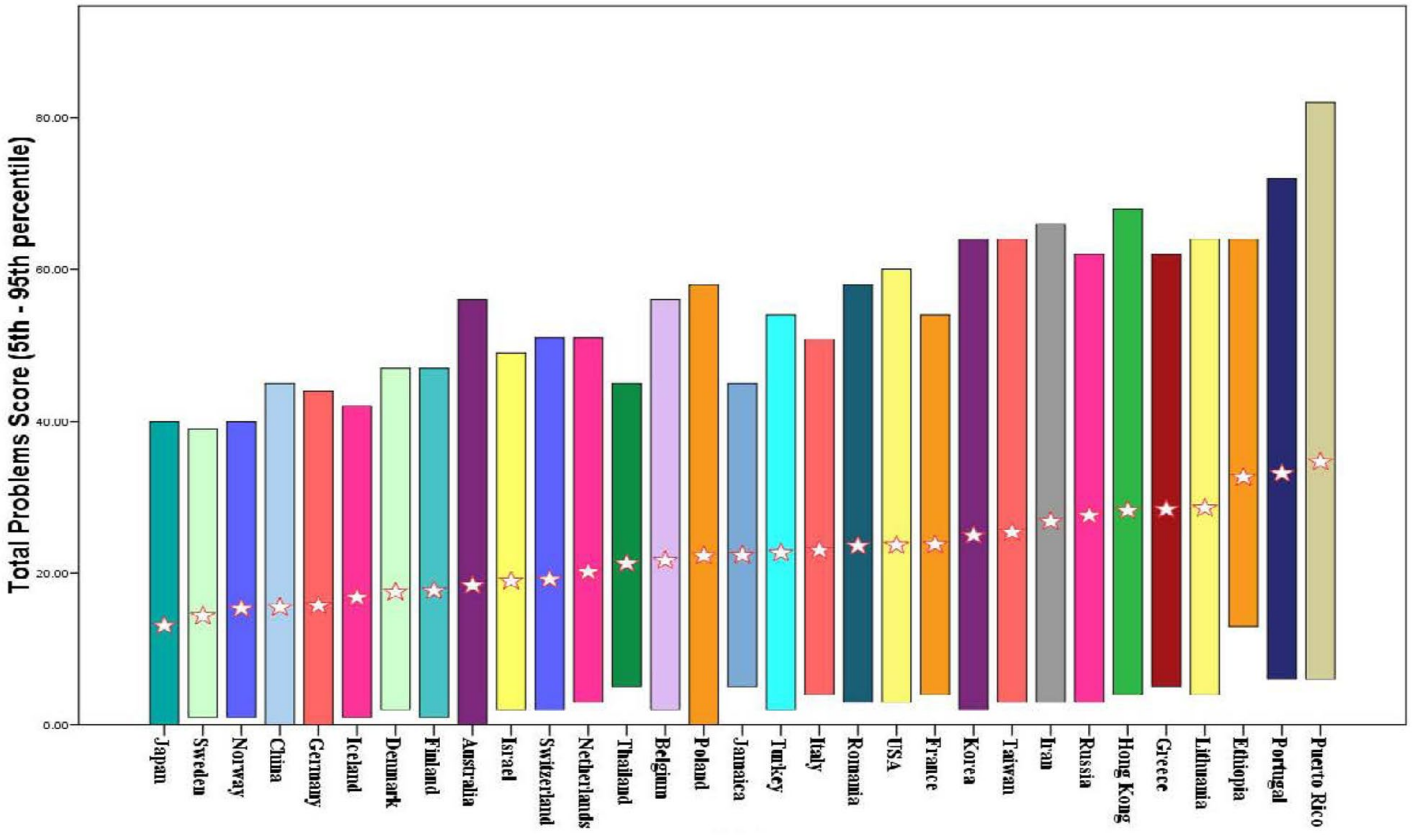
Distributions of CBCL/6–18 Total Problems scores: 5th to 95th percentiles. Stars indicate the mean Total Problems score for each society (from [1], p. 54)

Even though the patterns of co-varying problems embodied in the empirically derived syndromes were supported in dozens of societies, this does not necessarily mean that scores on the syndrome scales (sum of 0–1–2 ratings on the items comprising a scale) are similar in all societies. If the scores tend to be higher in some societies than in others, such differences need to be taken into account when assessing children in the different societies. To compare the magnitudes of problem scores across different societies, the mean Total Problems scores (sum of 0–1–2 ratings on all problem items on a form) were computed for population samples from each society. For a particular form—such as the CBCL/6–18, the mean Total Problems scores from all available societies were averaged to obtain the “omnicultural mean”, i.e., the mean of the mean Total Problems scores for all the available societies. Figure 1 displays bar graphs that span from the 5th to the 95th percentile CBCL/6–18 Total Problems scores in each of 31 societies.

The star in the middle of each bar indicates the mean Total Problems score for that society. Even though there were statistically significant differences between the scores for the different societies, the 5th to 95th percentile distributions for every society overlap with those for every other society. Thus, many children in Japan—the society with the lowest mean Total Problems score—obtained scores that overlap with scores obtained by children in Puerto Rico—the society with the highest mean Total Problems score. In other words, no society differed categorically from any other society in having scores that were all lower or all higher than in another society.

For each ASEBA form listed in Table 1, societies were identified whose mean Total Problems scores were more than one standard deviation (*SD*) below the omnicultural mean. These societies with relatively low problem scores on a particular form were designated as *Multicultural Group 1*. Scale scores from all the Group 1 societies were then combined to compute norms for each of the empirically derived syndromes. Norms were also computed for the Total Problems (general psychopathology) scale and for other scales scored from the ASEBA problem items. Other scales included DSM-oriented scales comprising problems identified by international experts as being very consistent with DSM-5 diagnoses [5], plus broad spectrum Internalizing and Externalizing scales [4].

**Fig. 2:**
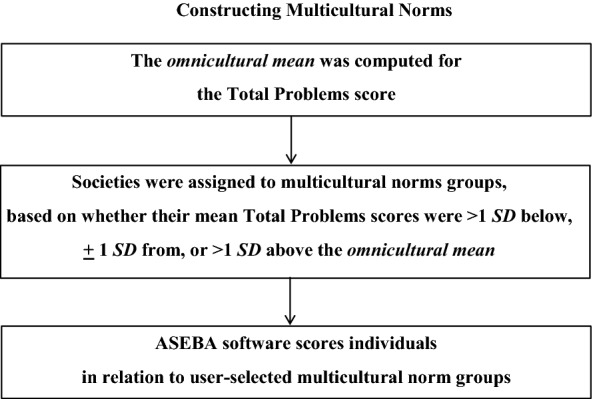
Procedures for constructing and applying multicultural norms (from [3])

In addition to the sets of multicultural norms for Group 1 societies, sets of multicultural norms were also constructed for societies whose mean Total Problems scores were > 1 *SD* above the omnicultural mean. These societies with relatively high problem scores were designated as *Multicultural Norm Group 3.* For some forms, the mean Total Problems score for the US normative sample was at the middle of the scores for the societies with mean Total Problems scores ranging from 1 *SD* below to 1 *SD* above the omnicultural mean. For those forms, the widely used US norms are used for *Multicultural Norm Group 2* societies. For other forms, Group 2 norms were constructed according to the procedures described for Group 1 and Group 3. Computer software for scoring ASEBA forms enables users to display scale scores in relation to Group 1, Group 2, or Group 3 norms, depending on the societies that are relevant to the person being assessed and the informants completing collateral-report forms. Figure 2 summarizes the procedures for constructing and applying multicultural norms.

The distributions of problem scores shown in Fig. 1 resemble the broad population distributions typically found for characteristics such as height and weight. The fact that societies differ with respect to their average problem scores, their average height, and their average weight means that those societal differences need to be reflected in norms for particular societies. Nevertheless, within each society, individual differences in problem scores, height, and weight must be identified to characterize each individual in the society. The ASEBA Multicultural Norm Groups enable users to separate societal effects from their assessment of individual children within societies.

In addition to societal effects, there may also be cultural effects that are not perfectly correlated with societal effects. However, hierarchical linear modeling analyses have shown that societal effects exceeded cultural effects and that the sum of societal effects plus cultural effects accounted for only about 10% of the variation in CBCL/6–18 scores obtained by 72,493 children living in 45 societies nested within 10 culture clusters (e.g., Anglo, Confucian) from every inhabited continent [23]. The finding that about 10% of the variation in problem scores is accounted for by societal and cultural effects means that most of the variation in problem scores is accounted for by effects associated with individual differences among children. In other words, most of the variation in CBCL/6–18 problem scores reflects differences among problems reported by parents for individual children within their societies and culture clusters.

### Applications to clinical services

Efforts to obtain help for children’s behavioral, emotional, social, and thought problems typically require information from adults, such as parents, caregivers, and teachers. The CBCL/1½–5 and CBCL/6–18 enable parents and others who see children in their home environments to provide ratings and personal comments on a broad spectrum of problems. Both forms also ask informants to describe what concerns them most about the child and the best things about the child. The CBCL/1½–5 includes the Language Development Survey, which can identify delayed speech. The CBCL/6–18 includes items for assessing competencies in terms of the child’s functioning in activities, social relationships, and school. The Caregiver–Teacher Report Form (C-TRF) enables preschool teachers and daycare providers to provide ratings and comments on many of the same problems assessed by the CBCL/1½–5, plus others that are more specific to group settings. The Teacher’s Report Form (TRF) enables teachers and school counselors to provide ratings and comments on most of the same problems as the CBCL/6–18, plus problems and adaptive functioning specific to school contexts. The Youth Self-Report (YSR) enables 11–18-year-olds to rate many of the same problems and competencies as are rated on the CBCL/6–18, plus the youth’s own positive qualities.

### Use of data from multiple informants

Most providers of child mental health services recognize that information is needed from multiple informants who can report on different aspects of a child’s functioning in different contexts. Differences between parent and teacher reports, for example, may reflect both differences in how a child functions at home versus school and differences in how the child is perceived by parents versus teachers. To help providers take account of the discrepancies that often occur between informants’ reports [8], ASEBA software displays bar graph comparisons between scores obtained from up to 10 informants for syndromes, DSM-oriented scales, Internalizing, Externalizing, and Total Problems. Scale scores are standardized on the basis of norms for the child’s age, gender, the type of informant (parent, teacher, self), and the relevant multicultural norm group.

#### Multicultural family assessment module (MFAM)

When parent figures are available, it is often as important to assess them as to assess the child who needs help. This can be done by asking each parent figure to complete the Adult Self-Report (ASR) to rate and report on their own problems and strengths. If more than one parent figure is available, each can also be asked to complete the Adult Behavior Checklist (ABCL) to describe their partner. The Multicultural Family Assessment Module (MFAM) is an app that can display bar graphs of ASR and ABCL scale scores alongside CBCL/6–18, TRF, and YSR scale scores. As seven ASR and ABCL syndromes have counterparts scored from the CBCL/6–18, TRF, and YSR, mental health providers can directly compare parent and child scores on the counterpart syndromes. In some cases, such comparisons may reveal similarities between parent and child problems, as has been found in US and Dutch studies [27, 29].

**Fig. 3:**
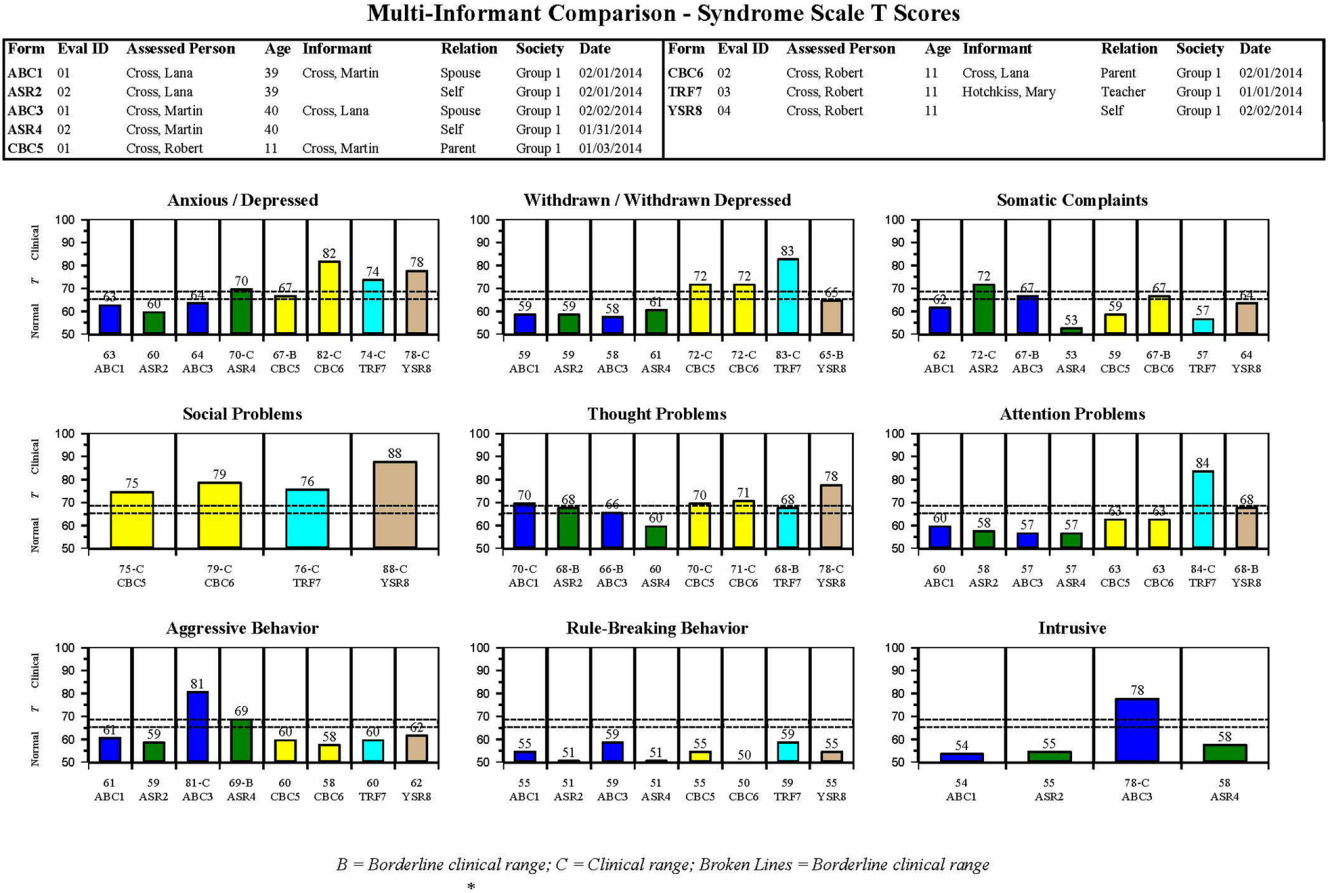
MFAM bar graphs of syndrome scores for Martin, Lana, and their son Robert (from [3])

As an example, Fig. 3 displays MFAM bar graphs for syndrome scales scored from ASRs completed by Martin and Lana to describe themselves, bar graphs scored from ABCLs completed by Martin and Lana to describe each other, and bar graphs scored from CBCL/6–18 forms completed by Martin and Lana to describe their 11-year-old son Robert, plus TRF and YSR forms completed to describe Robert (names and personal details are fictitious). By looking at the middle bar graphs in the middle row of Fig. 3, the provider can see that the Thought Problems syndrome scale scores are elevated for the ASR and ABCL that describe Lana, as well as for the CBCL/6–18, TRF, and YSR forms that describe her son Robert. The Thought Problems syndrome scale scored from the ABCL completed by Lana to describe her partner Martin also reached the bottom of the borderline clinical range (the bottom broken line in Fig. 3). These results provide evidence that Lana and her son Robert, and to a lesser degree Robert’s father Martin may be experiencing thought problems.

Other bar graphs in Fig. 3 indicate that Robert has elevated levels of problems of the Anxious/Depressed and Withdrawn/Depressed syndromes according to parent, teacher, and self-ratings, plus an elevated level on the Attention Problems syndrome scored from the TRF and a less elevated level on the Attention Problems syndrome scored from the YSR. On the Social Problems syndrome (not scored from the ASR or ABCL), Robert’s CBCL/6–18, TRF, and YSR forms all yielded scores in the clinical range (above the top broken line). On the Intrusive syndrome (scored only from the ASR and ABCL) and on the Aggressive Behavior syndrome, Lana’s ABCL ratings of her partner Martin yielded scores well up in the clinical range.

The mental health provider working with Robert, Martin, and Lana can elect to show the MFAM bar graphs to Martin and Lana to help them appreciate similarities and differences between how they see themselves and are seen by their partner. This may help them understand how perceptions of their son Robert may also differ and how problems reported for Robert may relate to their own functioning.

### Assessing progress and outcomes

Evidence-based practice entails obtaining explicit evidence about children’s functioning and needs when the children are initially assessed in order to design appropriate interventions. However, evidence-based practice should also include assessments to evaluate progress and outcomes. Assessments of progress should compare children’s functioning after interventions are implemented with their functioning at intake in order to determine whether functioning is improving. If not, changes in the interventions may be warranted. Assessments of outcomes should compare children’s functioning when interventions are ending with their functioning at intake in order to determine whether functioning has improved sufficiently to warrant ending services. If standardized assessment instruments are used to obtain data from multiple informants at intake, some or all of the same informants can be asked to complete the assessment instruments again in order to assess progress and outcomes.

To facilitate the assessment of progress and outcomes and to determine whether changes exceed chance expectations, the Progress & Outcomes App (P&O App; [2]) enables providers to compare ASEBA scale scores obtained at intake into a service with scores obtained at subsequent provider-selected intervals for progress and outcome assessments. The P&O App displays bar graphs of scale scores for each assessment, plus text statements regarding whether changes in scores exceed chance expectations, as determined by statistical criteria applied by the P&O App. Providers do not need any statistical skills to have the P&O App determine whether changes in scale scores for individual children exceed chance expectations. However, for providers, agencies, and researchers wishing to compare the effectiveness of different interventions with each other and/or with control conditions, the P&O App can also provide statistical analyses for comparing the progress and outcomes of groups receiving different conditions.

### Applications to research

ASEBA forms are widely used in research, with over 10,000 publications reporting their use in over a hundred societies and cultural groups [7]. Research applications of ASEBA forms include epidemiological studies of the prevalence and patterning of problems in many societies, as exemplified by the Rescorla et al. [23, 24] studies of problems reported for population samples of children in dozens of societies.

ASEBA forms are especially well suited to research that requires re-assessments of children over long periods, such as studies of the outcomes and effectiveness of particular interventions and longitudinal studies of the developmental course, correlates, and outcomes of diverse problems and strengths. Because ASEBA forms include developmentally appropriate items, scales, constructs, and norms for ages 1½–90 + years, the same individuals can be repeatedly assessed with ASEBA forms as they advance through successive developmental periods. Moreover, the standardization of ASEBA data across developmental periods facilitates statistical analyses for identifying continuities and changes in individuals’ functioning as they develop.

Examples of longitudinal studies employing ASEBA assessments that have yielded many findings on the developmental course, correlates, and outcomes of diverse problems and strengths include the US National Longitudinal Study of a representative sample of over 2000 US children assessed over 9 years into early adulthood [28]; the Zuid Holland Longitudinal Study of over 2000 Dutch children assessed over 24 years into middle adulthood, when the original participants’ children were also assessed [22, 26]; the TRacking Adolescents Individual Lives Survey (TRAILS) of Dutch adolescents, including a population sample of over 2000 youths and a clinical sample of over 500 youths [20]; the Generation R Study (“R” = Rotterdam) that started with 8880 pregnant women [25]; and the Netherlands Twin Registry that has assessed twins born in the Netherlands each year since 1987 and has re-assessed them as they developed into adulthood [10].

Among the many studies generated by the Netherlands Twin Registery is one that estimated genetic and environmental variance in scores on the CBCL/1½–5 Pervasive Developmental Problems scale (“Autistic Spectrum Problems” scale since DSM-5 was published) [9]. Based on data for 38,798 3-year-old twins, genetic effects accounted for 78% of the variance in boys’ scores and 83% of the variance in girls’ scores. Nevertheless, 29% of monozygotic twins were discordant for clinical versus normal range scores, suggesting that environmental factors might provide resilience for some children, despite high genetic risk.

ASEBA forms are widely used to test the effects of interventions in randomized clinical trials (RCTs), where children receiving different intervention and control conditions are assessed with ASEBA forms at intake and again following the intervention conditions. As an example, computerized cognitive training was provided to randomly selected Ugandan children who had survived cerebral malaria, while a randomly selected control group did not receive training [6]. Before and after the training periods, parents or surrogates completed the CBCL/6–18 and the children received six cognitive tests. The intervention group improved significantly more than the control group on the CBCL/6–18 Internalizing scale and on 3 of the 6 cognitive tests and nonsignificantly more on the CBCL/6–18 Externalizing and Total Problems scales, as well as on the other three cognitive tests. The authors concluded that the training could improve the behavioral and cognitive functioning of children who had survived cerebral malaria.

As another example, an RCT of an omega-3 dietary supplement for children in Mauritius was followed by significantly lower CBCL Internalizing and Externalizing scores for children receiving omega-3 than for children receiving a placebo [21].

### Applications to training

Mental health trainees can learn the value of obtaining and comparing evidence from parent-, teacher-, and self-reports by working with children for whom the CBCL, C-TRF, TRF, and/or YSR are completed. Trainees can study a completed CBCL before interviewing a parent or a completed YSR before interviewing a youth and can then ask the interviewee if they have any questions about the form. This often elicits responses that provide leads regarding the respondent’s concerns. Trainees can also ask about items that were endorsed on the form. For example, if a parent gave a *1* or *2* rating to *Can’t get mind off certain thoughts* and wrote “death” in the space that invites a description of the problem, the trainee can mention the parent’s response and ask the parent to talk about it. If a youth gives a *1* or *2* rating to the YSR item *I feel that others are out to get me*, the trainee can ask the youth to talk about it. Parents and youths often report many more problems on the CBCL and YSR than they would spontaneously volunteer in interviews.

By viewing comparisons of CBCL, C-TRF, TRF, and/or YSR item and scale scores that are displayed by ASEBA software, trainees can identify specific consistencies and discrepancies between reports by different informants. Trainees can thus identify problems likely to warrant a broad-gauged intervention because they are reported by all informants versus problems that may warrant a more situation-specific approach because they are reported to occur in only one context, such as home or school. Other problems may be specific to interactions with only one informant, such as one parent or one teacher.

If parents are asked to complete the ASR to describe themselves and to complete the ABCL to describe their partner, the MFAM can be used to display bar graphs of scores obtained from the ASR and ABCL alongside bar graphs of scores obtained from the CBCL/6–18, TRF, and/or YSR. By comparing the parent and child scores, trainees can identify similarities and differences between their scores as an aid to formulating intervention plans and deciding whether to show the MFAM output to parents. After trainees are acquainted with the parents and child, they can also fill out ABCL and CBCL forms for comparison with the forms completed by family members. To sharpen their clinical skills, trainees can then discuss discrepancies between the trainee-completed forms versus the parent-completed forms with the trainees’ supervisors. After interventions have been implemented, parents and/or youths can be asked to complete the forms again to evaluate progress and outcomes. If trainees (blind to the forms completed by family members) then complete the relevant forms, they can have ASEBA software compare them with the results obtained from family members to sharpen their skills for evaluating progress and outcomes.

## Summary and conclusions

This article presented multicultural norms and related international findings obtained via standardized forms for ages 1½–90+ years by collaborating indigenous researchers in over 50 societies from every inhabited continent. Based on assessment of population samples, the multicultural norms enable mental health providers to display individuals’ scores for syndromes, DSM-oriented scales, Internalizing, Externalizing, and Total Problems in relation to norms for the assessed person’s age, gender, the type of informant who provided assessment data, and the appropriate multicultural norm group.

Because children’s functioning often differs from one context to another—such as home versus school—and because perceptions of children also differ, it is essential to obtain data from multiple informants, such as a child’s mother, father, teacher(s), and the child. Parallel assessment forms designed for completion by parents, teachers, and youths are scored via software that displays side-by-side comparisons of item and scale scores. Providers can thus identify consistencies and discrepancies between reports by different informants to consider in planning interventions.

Because parent figures play key roles in efforts to help children, self- and collateral-report forms for parents can be used to document and compare parents’ functioning with their children’s functioning. Evidence-based practice entails obtaining explicit evidence regarding functioning at intake into services and again on subsequent occasions to assess progress and outcomes, which can be done with the Progress & Outcomes App.

Applications to clinical services, research, and training were presented to demonstrate the value of using the same standardized assessment instruments for many purposes in diverse populations around the world.

## Limitations and future directions

The ASEBA provides practical instruments for the phenotypic assessment of psychopathology and strengths, based on self- and collateral-reports, scored from a finite set of items. Although respondents are encouraged to describe additional problems and strengths, different items and analyses may well produce different results. Developmental histories, interviews, observations, and biomedical procedures also contribute to comprehensive assessment. Moreover, genetic, behavioral, neurobiological, and other research methods are essential for advancing knowledge of influences on the phenotypic psychopathology and strengths assessed by the ASEBA.

For the future, multicultural collaborations on evidence-based assessment will continue to expand beyond the 50+ societies from which indigenous collaborators have contributed data. A key objective is to disseminate evidence-based assessment tools, attitudes, and practices in order to ensure that initial evaluations provide data with which to optimize interventions and against which to measure changes at subsequent progress and outcome assessments.

## Data Availability

No datasets were generated or analyzed for this article

## Abbreviations

CBCL/1½–5 and CBCL/6–18: Child Behavior Checklist
CFA: confirmatory factor analysis
DSM: diagnostic and statistical manual
TRF: Teacher’s Report Form
YSR: Youth Self-Report
MFAM: Multicultural Family Assessment Module
ASR: Adult Self-Report
ABCL: Adult Behavior Checklist
P&O App: Progress & Outcomes App
RCT: randomized clinical trial
C-TRF: Caregiver–Teacher Report Form
